# Puma (*Puma concolor*) in the Neighborhood? Records Near Human Settlements and Insights into Human-Carnivore Coexistence in Central Chile

**DOI:** 10.3390/ani11040965

**Published:** 2021-03-31

**Authors:** Diego Ramírez-Álvarez, Constanza Napolitano, Iván Salgado

**Affiliations:** 1 Unidad de Vida Silvestre, Servicio Agrícola y Ganadero (SAG), Región de O’Higgins, Rancagua 2820000, Chile; ivan.salgado@sag.gob.cl; 2 Departamento de Ciencias Biológicas y Biodiversidad, Universidad de Los Lagos, Osorno 5312435, Chile; 3 Instituto de Ecología y Biodiversidad (IEB), Santiago 7800003, Chile

**Keywords:** wildland–urban interface, human–carnivore coexistence, human-dominated landscapes, O’Higgins region

## Abstract

**Simple Summary:**

The presence of carnivores near human settlements is a poorly studied topic that generates concern and perception of risk in some human communities, especially for medium to large felids. Apart from the conflict of the potential predation of livestock, there is the insecurity perception of a potential attack on people. To gain a better understanding of how, when, and how close pumas approached human settlements, we analyze 51 puma records near populated areas over eight years in central Chile. The results show that pumas approached human-populated areas; in 23.5% of the records pumas are found between 0 and 999 m from the nearest human settlement, 25.5% are between 1000 and 4999 m, and 51% are over 5000 m. We associate puma records with landscape features, such as mountain ranges, land-use, road, and urban infrastructure; and based on previous knowledge of puma biology, behavior, and habitat preference, we identify their area of occupation and the potential biological corridor used for their movements from the Andes Range to the coast. Our results show the adaptability of pumas to human-dominated landscapes, and their capacity to overcome landscape barriers, such as human infrastructure, contributing to a better understanding of the population dynamics in the study area. Studies on human–carnivore coexistence, through formulas that consider local realities and the reduction of implicit risks for humans, are urgently needed, both globally and locally, and likely the only way to secure the long-term conservation of pumas in human-dominated landscapes.

**Abstract:**

The wildland–urban interface lies at the confluence of human-dominated and wild landscapes—creating a number of management and conservation challenges. Wildlife sightings near human settlements have appeared to increase in the last years. This article reports 51 records of presences, sightings, and livestock attacks of *Puma concolor*, a large-sized felid, collected from 2012 to 2020 across the O’Higgins region in central Chile. Puma records were concentrated in the east of the region in the Andes Range and foothills (90%). The number of puma records is higher in the last four to six years than in previously studied years. Of the 51 records, 23.5% are between 0 and 999 m from the nearest human settlement (classified as very close), 25.5% are between 1000 and 4999 m (moderately close), and 51% are over 5000 m (distant). Most of the sightings are recorded in the summer (35%) and spring (29%). We identify an area of approximately 9000 km^2^ of suitable habitat as the most probable corridor effectively connecting pumas moving between eastern and western areas, encompassing the Angostura de Paine mountain range. Our results contribute to the understanding of the presence and movements of *P. concolor* near urban areas and human settlements, confirming their persistence in and adaptation to human-dominated landscapes. We also provide insights into human–carnivore coexistence in the current global context in the densely populated central Chile.

## 1. Introduction

*Puma concolor* [1] (puma, cougar, mountain lion) has a wide latitudinal range of distribution in the American continent [2,3], from southeast Alaska [4] to the austral Patagonian plains in Chile and Argentina [5,6]. This species has high dispersal capacity, being capable of covering large distances in short periods of time [7,8,9,10,11]. *P. concolor* home range size varies by sex, age, season, and spatial distribution and density of prey [12,13,14,15].

Home ranges between 65 km^2^ and 510 km^2^ have been reported in North America. Home ranges of resident male mountain lions are typically larger than those of females; they overlap a number of female home ranges, but only occasionally those of other resident males. The mean home range for resident males is between 437 and 510 km^2^; for resident females, it is between 177 and 192 km^2^ [16,17].

The Chilean subspecies, *P. concolor puma* [18] [3], is distributed in the country from the border with Peru as the northern limit to the Strait of Magallanes in the south, excluding Chiloé Island and Tierra del Fuego [6,19].

Home ranges in Chilean Patagonia varied between 24 and 260 km^2^; female home ranges extensively overlapped with those of other males and females, but male home ranges overlapped for only short time periods [20,21]. In the Coquimbo region of northern Chile, home ranges of 503 km^2^ and 631 km^2^ were reported for an adult female and a male, respectively, the largest home ranges recorded for the species in Chile [22]. These studies confirm that home ranges are inversely correlated with prey density [21].

The current spatial occupation of the species in central Chile is closely associated with the Andes Range [23,24]. However, the species can occupy a wide diversity of ecosystems, including desert, tropical forest, Altiplano, temperate forests, coastal plains, and Mediterranean scrubland [25,26,27]; its pre-Hispanic distribution in central Chile included the entire altitudinal territory, from sea level to the high mountains [28,29,30].

**Figure 1:**
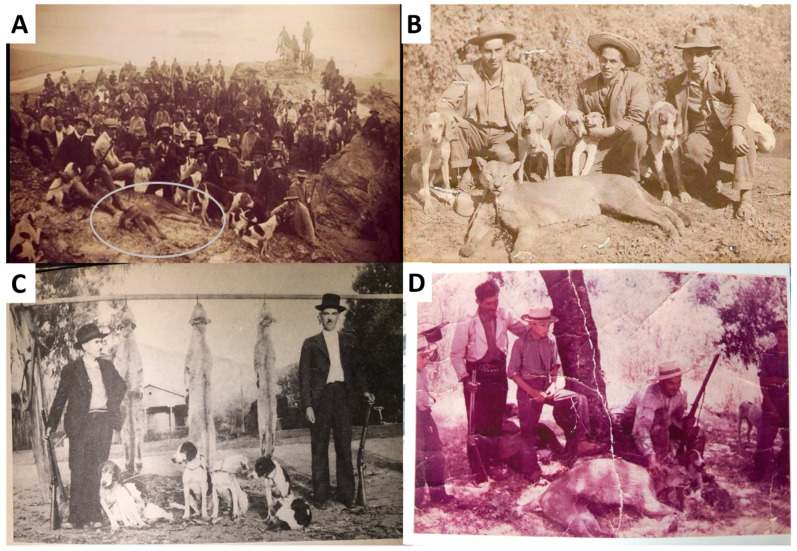
Historical photographs of people hunting *Puma concolor* in O’Higgins region, central Chile. (**A**) A massive hunt of pumas and foxes organized by a cattle ranch near Pichilemu, 1923, in the Coast Range. (**B**) From 1947, Altos de Cantillana, a mountain range in the intermediate depression. (**C**) From 1951, “leoneros”, puma-specialized hunters, in the Andes foothills in central Chile. (**D**) From 1966, Loncha, a mountain range in the intermediate depression, central Chile.

According to the IUCN Red List, the current conservation status of pumas for their complete distribution range is Least Concern [31], while the national classification in Chile is Near Threatened [32]. Among the factors that have been reported to facilitate a sustained population decline of pumas in Chile are the historical modification of its habitat, indiscriminate hunting in past decades (Figure 1), and a drastic reduction of its natural prey [33]. In fact, given its generalist feeding habits, in central Chile, the puma has had to modify its diet from the ancestral consumption of native prey, mainly *Lama guanicoe*, to post-colonization introduced prey, mainly lagomorphs *(Oryctolagus cuniculus and Lepus capensis)* and to a lesser extent domestic cattle [34,35].

Central Chile has experienced a notable expansion of the human-population into wilderness areas in recent decades, converting land to residential or agricultural use [36,37]. Together with the transhumance activity of cattle or mountain range summer grazing, this has led this felid to have greater availability of domestic prey, such as goats, sheep, cattle, and horses, accentuating the historical conflict between it and livestock production [35,38,39,40].

The O’Higgins region, an administrative territory in central Chile, encompasses an area of 16,387 km^2^ of the rich Mediterranean ecosystem and is part of the 25 priority hotspots for the conservation of global biodiversity [41]. This region presents the representative geographical topography of central Chile from east to west: Andes Range, Andes foothills, intermediate depression, Coast Range, and coastal plain. 

Records of permanent puma presence in the coastal area and intermediate depression of the O’Higgins region in past decades were compiled by Castillo [29]. Unfortunately, the historical persecution and hunting by humans in these areas (Figure 1), due to conflict with livestock activities and occupation for human settlements and agricultural activity, relegated the species currently mostly to the Andes Range area of the region. 

*P. concolor* maintains a stable population with a density of 0.75 adults per 100 km^2^ in the Alto Cachapoal basin [42] in the Andes Range of the O’Higgins region, relatively far away from human activity. This is one of the lowest densities for the species in South America, compared to densities of 3.4 to 6 individuals per 100 km^2^ reported for Chilean Patagonia [10,20] and 3.4 to 6.8 individuals per 100 km^2^ reported for the tropical forests of Brazil and the Bolivian Chaco, respectively [43,44]. However, this small Andean population in the O’Higgins region shows healthy ecological and biological dynamics, apparently breeding successfully, producing offspring that successfully reach the age of dispersal and adulthood, and feeding mainly on lagomorphs, birds, and other native mammals, and to a much lesser extent on transhumant cattle during the summer season [35,45]. 

The species has been banned from hunting and capture in Chile since the early 1980s, which may have facilitated a relative recovery and stabilization of its populations. Reports of cougar sightings and/or attacks on domestic cattle are increasingly frequent in recent years in the lower foothills, the intermediate depression, and even in the coastal area of the O’Higgins region [46].

Here we report records of *P. concolor* from the last eight years (2012–2020) across the O’Higgins region in central Chile, showing their distance of approach to human settlements, proposing suitable habitat areas for spatial movements and dispersal, and discussing human-wildlife coexistence in the current global context. We document that this species, which we believe commonly displays refractory behavior towards humans and mostly occupies territories far from urbanization, in some situations approaches human settlements.

## 2. Methods

The Agricultural and Livestock Service (SAG, for its acronym in Spanish), part of the Ministry of Agriculture, is the Chilean state institution in charge of the administration and supervision of hunting law regulations and protecting native species in the country. Between May, 2012, and July, 2020, SAG from the O’Higgins region received (citizen’s reports) and collected from its own sources records of presence, sightings, and livestock attacks of *P. concolor* in different locations across the region. Only verified species assignments were included in this study: Photographs, video recordings, trail camera records, corpse findings, live captures for research purposes, and forensic analysis of attacked cattle. The following data were obtained for each record: Location, date, georeference, altitude, and distance to the nearest human settlement calculated from its perimeter. The settlement was defined as a permanent dwelling site inhabited by more than 100 people. To assess the distance of approach to human settlements, the spatial data was analyzed using the ArcGIS-Pro^®^ software, and a digital elevation model (DEM) obtained from the PALSAR (Phased Array Type L-band Synthetic Aperture Radar) sensor of the Japanese satellite ALOS (Advanced Land Observation Satellite) with a sampling resolution of 12.5 m (Hi-Res Terrain Corrected), along with a cartography of the road network and populated areas in the region. We classified the distance from the *P. concolor* record to the closest human settlement in three categories: (a) Very close, when the distance was between 0 and 999 m; (b) moderately close, the distance between 1000 and 4999 m; and (c) distant, over 5000 m.

To identify suitable habitat probably used by pumas to move across the region and explore the potential corridor between east and west, we conducted a spatial analysis using ArcGIS Pro^®^ combined with the Chilean land cover layer (LandCover-2014), a land-use vector layer developed by the National Institute of Statistics of Chile (INE for its acronym in Spanish), and a high-resolution digital elevation model from the ALOS PALSAR sensor. Landscape features suitable for puma movements (habitat) were identified and included as geographic inputs: Continuity of vegetation cover, wooded or shrub areas, rugged terrain, and abrupt changes in elevation. We excluded roads and areas of high urban anthropic activity (following puma habitat suitability criteria; [47,48,49]. Landscape features were identified through two digital cartographic tools: The Digital Elevation Molding (DEM) of the Alos Palsar sensor, with a native resolution of 12.5 m, and the Land Cover Classification System (LCCS), a product that discriminates land cover between its biological and physical components, at a spatial resolution of 30 m with at least three levels of classes. To identify and exclude urban areas with high anthropic activity, we used a digital cartographic layer of geographical census division, which discriminates between areas of urban demographic concentration, and other human settlements with low population concentration. 

## 3. Results

**Figure 2:**
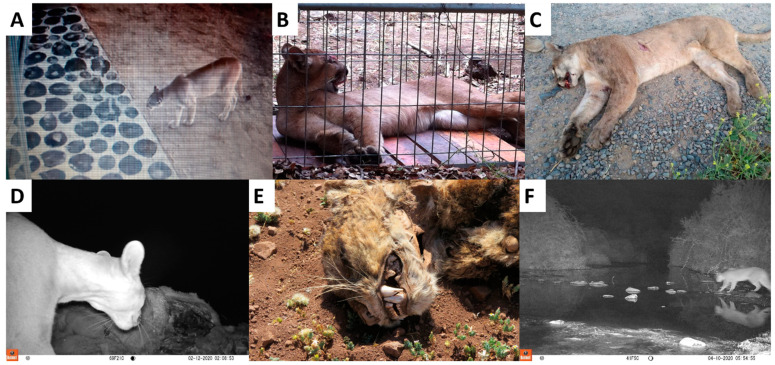
Records of *P. concolor* across the O’Higgins region in central Chile (selection). Different types of evidence are shown: Security cameras (**A**), trail camera photographs (**D**,**F**), live captures (**B**), and corpses found (**C**,**E**). Letters correspond to sites referenced in Figure 3.

**Figure 3:**
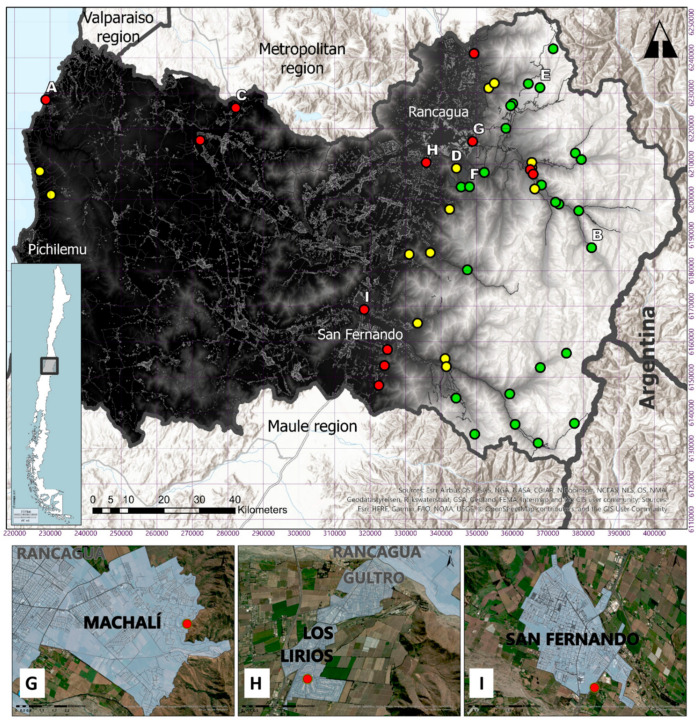
The geographic location of records of *P. concolor* in the O’Higgins region, central Chile. Color coding according to proximity to human settlement: Red circle—very close (0 to 999 m); Yellow circle—moderately close (1000 to 4999 m); Green circle—distant (>5000 m). Letters correspond to records from Figure 2 and three sites indicated at the bottom of the figure (G: Machalí, H: Los Lirios, I: San Fernando). Below: The three closest records within the urban limits of large cities in the O’Higgins region. Grey shadow: Urban area, darker grey = high demographic concentration, lighter grey = low demographic concentration.

We collected 51 records of the presence of *P. concolor* across the study area (Table 1, Figure 2). Puma records were concentrated in the east of the region, in the Andes Range and foothills (46/51, 90.2%). Only five records (5/51, 9.8%) occurred in the western area, in the intermediate depression, Coast Range, and coastal plain (Figure 3).

**Figure 4:**
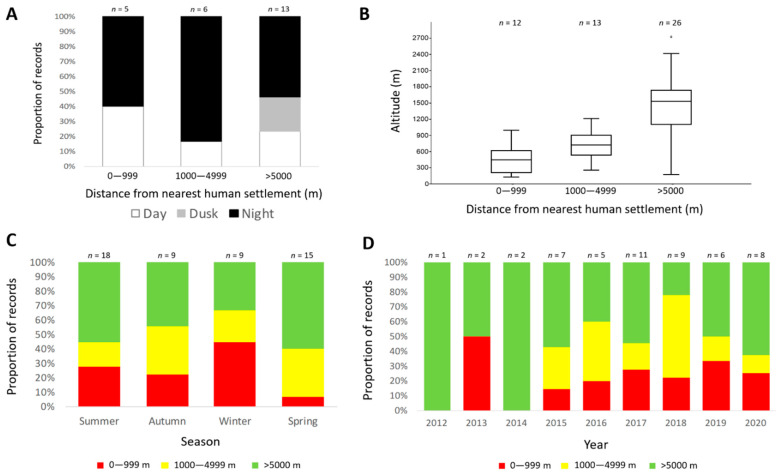
*P. concolor* records in relation to distance from the nearest human settlement (m), (**A**) time of the day, (**B**) altitude, (**C**) season, and (**D**) year of the study period. Color coding according to proximity to human settlement: Red—very close (0 to 999 m); Yellow—moderately close (1000 to 4999 m); Green—distant (>5000 m). Dot above box plot in (**B**) corresponds to an outlier data point.

Twelve (23.5%) of the 51 records were between 0 and 999 m from the nearest human settlement (classified herein as very close), 13 (25.5%) were between 1000 and 4999 m (moderately close) and 26 records (51%) were over 5000 m from the nearest human settlement (classified as distant) (Figure 3 and Figure 4).

Records varied from 132 to 2725 m elevation, with a median of 465 m for the records very close to human settlements, and 724 and 1471 m for records moderately close and distant, respectively. We found statistically significant differences between distance classes and altitude (Figure 4B) for all comparisons (very close-moderately close: U = 39, z = 2.09, *p* = 0.037; moderately close-distant: U = 39.5, z = 3.84, *p* = 0.00012; very close-distant: U = 18, z = 4.32, *p* < 0.00001).

Three of the 12 records classified as very close to human settlements occurred within the urban limits of cities: Los Lirios in Requínoa (27,968 inhabitants), Machalí (52,505 inhabitants), and San Fernando (58,367 inhabitants) [50] (Figure 3). In two of these records, through security cameras pumas were observed crossing the garden of inhabited houses, and in one of them, the animal is looking into a house through the window in a stalking position, probably due to an indoor pet (Figure 2A).

Almost all records classified as very close to human settlements corresponded to *P. concolor* in transit, where the animal was not seen again prowling in the area, therefore no capture or translocation was required, except for one case in Machalí (Figure 3G). In this case, a cougar was cornered by a group of stray dogs (*Canis familiaris*) among dense *Rubus ulmifolius* scrub in an urban park; therefore, a containment operation had to be conducted to restrain the dogs, and a controlled escape route towards the nearby hills had to be secured for the puma, through which it finally escaped into its natural habitat.

The human settlement that showed the highest recurrence of *P. concolor* sightings, with two very close records and three moderately close records, was Chacayes (276 inhabitants, 34°14′59′′ S 70°28′30′′ W, 917 m) [50], an Andean village in the Machalí commune at the junction of the Pangal and Cipreses Rivers and at the entrance of the Rio Cipreses National Reserve.

All records classified as distant from human settlements corresponded to sightings in the Andes Range and its foothills, either in mining or hydroelectric facilities, derived from studies with camera traps, or occasional findings in hiking or mountaineering activities. Three of the five records located in the western area were very close to human settlements, and the other two moderately close (Figure 3). Two of them were located in the intermediate depression, a finding of a puma corpse (killed by a vehicle collision) near the town of Santa Inés and a sighting in Lago Rapel, in addition to three other sightings in the Coast Range pine plantations and the coastal plain.

Most of the sightings were recorded in the summer and spring seasons; 18 in summer (35%), 15 in spring (29%), 9 in autumn (18%), and 9 in winter (18%), and mainly in December (Figure 4C, Table 1). The season with the highest proportion of occurrence of very close records was winter (44%), and that with the lowest proportion was spring (7%) (all comparisons statistically non-significant).

The number of records was higher for the last 4 to 6 years (2015–2020) compared to the number of sightings recorded in the previously studied years (2012–2014) (Figure 4D, Table 1). From 2015 onwards, a higher proportion of occurrence of very close and moderately close sightings was recorded (all comparisons statistically non-significant).

Only 16 of the 51 records had photos (12 records) or videos (four records). In these images we could identify four males (25%, 4/16), six females (37.5%, 6/16) and six juveniles (37.5%, 6/16). Two records corresponded to a female with two cubs. The estimated body condition for the animals in these images were: Three males 3/5, one male 4/5, six females 3/5, four juveniles 3/5, and two juveniles 2/5.

Only 24 of the 51 records had confirmed the time of the day when the sighting occurred. For puma records very close to human settlements, 40% (2/5) occurred during the day and 60% (3/5) at night. For moderately close records, 17% (1/6) occurred during the day and 83% (5/6) at night. For distant records, 23% (3/13) occurred during the day, 23% (3/13) at dusk, and 54% (7/13) at night (Figure 4A) (all comparisons statistically non-significant).

**Figure 5:**
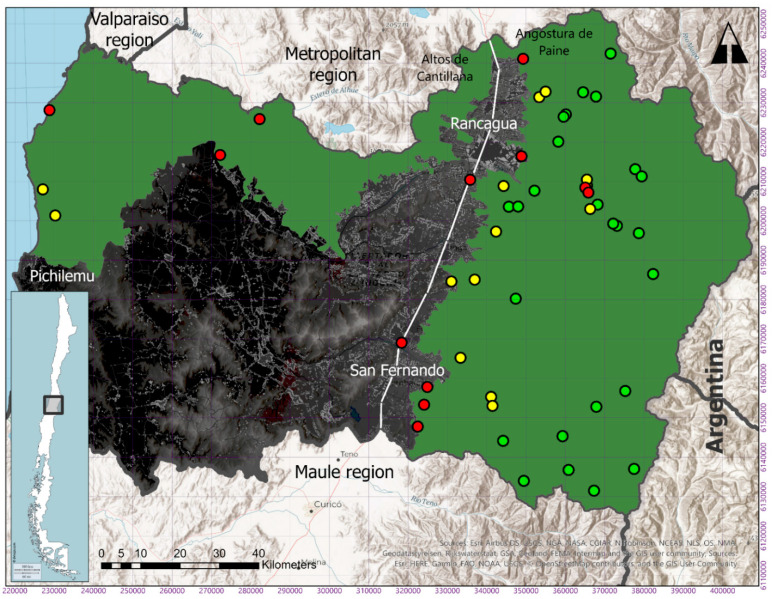
Suitable habitat for *P. concolor* movements across the region and most probable corridor between eastern and western areas. An area of approximately 9000 km^2^ of suitable habitat was estimated (in green). The mountains of Angostura de Paine and Altos de Cantillana were identified as the most probable corridor for east-west puma movements. White line represents the main Chilean north-south highway (Ruta 5 Sur). Map scale 1–700,000.

The spatial analysis to identify suitable habitat probably used by pumas to move across the region estimated an area of approximately 9000 km^2^, the most probable corridor east-west being the mountains of Angostura de Paine to Altos de Cantillana, which range across the north of the region (Figure 5). Based on our records, this mountain range could effectively connect pumas between the eastern and western areas.

## 4. Discussion

The records presented here are relevant to understand the presence and movements of *P. concolor* near urban areas and human settlements. They confirm the persistence of the species in the intermediate depression and coastal area of central Chile, overcoming urban infrastructure and adapting to human-dominated landscapes that could potentially act as barriers to their dispersal [51]. 

Obtaining evidence of the presence of *P. concolor* in the intermediate depression of central Chile is difficult, even with a good sampling effort. Recently, Garcia et al. [52] studied the presence of carnivores for four years (2013–2016), installing 53 camera traps (baited with lynx urine) in 12 remnant patches of sclerophyllous forest and shrubland in vineyard landscapes of the Mediterranean region of central Chile, but did not record *P. concolor.*

In this study, records classified as very close and moderately close were concentrated in the transition zone between the Andes foothills and the intermediate depression, parallel to the main Chilean north-south highway (Ruta 5 Sur), where most of the populated areas are located. This may explain the significant differences found between distance classes and altitude, and not an actual behavioral difference between the Andes foothills and the lowlands.

Detection probability may not be homogeneous across the study area, and may be higher near human settlements. We acknowledge the observed pattern of distribution may be biased accordingly, and this should be considered when interpreting the results. In this study, we did not find a higher number of records closer to human settlements. 

Puma records concentrated along the east of the main Chilean north-south highway, where most of the urban infrastructure is located, suggests that this densely populated strip may constitute one of the determining filters in the dispersal of the species from the east (Andes) to west (coast). It has been described that anthropogenic obstacles modify landscape permeability for pumas, and that dispersal distances are shorter in fragmented than in continuous landscapes [53]. At the wildland–urban interface, Kertson et al. [54] also found that early-successional forest (+), conifer forest (+), distance to the road (−), residential density (−), and elevation (−) were significantly positive and negative predictors of habitat use for *P. concolor*.

The locality of Chacayes, where the highest recurrence of sightings of *P. concolor* was recorded, is immersed in an area previously determined as of high transit of *P. concolor* through studies with satellite positioning collars [35]. Small livestock farming, the main economic activity, together with its geographical location in the Andes foothills may influence this greater number of sightings. Coincident with historical *P. concolor* sightings and information, we found no records in the southwestern part of the region, which has been the case for at least the past seven decades (personal communications of local inhabitants).

The coastal records of *P. concolor* in the region support previously studies in Chile reporting that pumas are habitat and diet generalists [48]. In the coastal area, forest plantations of exotic species (*Pinus radiata*) cover extensive surfaces, providing adequate vegetation coverage for its lurking predation habits and abundant populations of lagomorphs, which together with the occasional consumption of sheep may constitute its local main diet.

Considering that some puma reproductive behavior (communication and denning) may be affected by the proximity to urbanized areas [55], it remains to be clarified whether the coastal and intermediate depression sightings recorded in this study correspond to resident puma subpopulations or to non-resident animals bi-directionally transiting animals coming from the Andean zone. In a metapopulation framework, puma subpopulations separated by areas of non- or less-suitable habitat could be connected by dispersers, providing gene flow [47,56]. The ecological flexibility of pumas and their high dispersal capabilities can promote wide genetic connectivity across large geographic areas [57].

The proposed east-west corridor may connect from the Andes Range and its foothill, to hill chains in the Angostura de Paine sector, Altos de Cantillana and its southern mountain range, and to the Coast Range and coastal plains in the west, at least to the town of Pichilemu in the south. The east-to-west dispersal of *P. concolor* in the region may follow the source-sink model of population dynamics, which predicts that density drives emigration of subordinate animals to habitats offering lower competition for resources [7], considering that the decision of whether or not to disperse is multi-factorial, context-dependent and highly individualistic [58]. 

The dispersal of pumas is driven mainly by the search for food and reproductive partners in adults within their territories [10,11]. During the dispersion period of juveniles (between 1 to 2 years of age), the mother expulses her descendants from their birth territory, inducing them to search for their own [56]. It is mainly in this period when young inexperienced individuals may approach populated areas in search of food or to establish their own territories, and are subjected to the pressure of not being able to settle down or being expelled from territories already occupied by adult pumas. In this study, 37.5% of all puma records with identified sex were juveniles.

The proportion of occurrence of records in the different distance classes varied across the temporal distribution (year, season, and time of the day). However, all comparisons were statistically non-significant, probably due to the low sample size. The season with the highest proportion of very close records was winter, probably when snow and food scarcity forces some individuals to descend to lower elevations. The season with the lowest proportion of very close records was spring, with high prey availability for pumas [59].

Regarding the time of the day when the sightings occurred, very close, moderately close, and distant records had an approximately similar proportion of occurrence during dusk/night (60% night, 83% night, and 77% dusk/night (23% dusk, 54% night), respectively), assuming both dusk and night may facilitate puma movements being undetected by humans. However, we cannot properly assess this with our limited dataset. In other large felids, such as leopards, it has been described that in human-dominated landscapes animals shift their activity patterns towards markedly nocturnal [60].

The increased number of puma records we report here in the last 4 to 6 years compared to previously studied years is most probably due to a sustained increase in awareness of the general public and use of social media to share wildlife sightings, especially near human settlements. A growing interest of citizens in wildlife watching, new technologies for observing and capturing images, such as camera traps and the ease of sharing records through social networks, increasingly shows that it is not unusual for carnivores to move through urban areas [61,62,63]. However, this trend may also be attributable in part to a real increase in puma presence/abundance in the region, due to greater protection for the species, however, with our current data, we cannot assess this. Both of these factors may be operating, and other variables, such as yearly climatic conditions and prey availability, could also influence this trend. SAG has not changed its monitoring protocols, database management, or increased surveillance efforts during the period studied.

An unusual increase in puma sightings within the urban radio occurred during 2020 in the capital city of Santiago in Chile (Metropolitan region, adjacent to our study area), attracting the attention of the general public and especially of the wildlife authorities (SAG), who had to capture and relocate the animals. Among possible causes that have been proposed are factors, such as the decrease of prey in the Andean area, due to a long-term mega-drought, the deregulated expansion of the real estate industry into wild areas, and the lockdown effect during the Covid-19 pandemic [64]. A source-sink model of population dynamics from the more abundant source populations in the Andes Range close to the city of Santiago may be another possible cause [7]. Some carnivores approach cities profiting from resources generated by humans or even establish their ecological niche in cities [65,66,67]. However, urban wildlife ecology studies, especially in carnivores, are scarce in South America [63,68]. Therefore, all these possible explanations should still be considered as hypotheses under analysis and evaluated with caution in an inter-disciplinary approach [63].

The small sample size in this study limited the possibility of addressing potential statistically significant patterns on the relationship between the distance to human settlements of puma records and other variables, such as year, season, time of the day, sex, age group, or body condition. Future studies should aim at collecting a larger, long-term dataset covering a much wider area to elucidate many of the aforementioned unsolved questions.

The wildland–urban interface lies at the confluence of human-dominated and wild landscapes, creating a number of management and conservation challenges [54]. For example, the approach of *P. concolor* to human settlements and urban areas may facilitate a higher probability of indirect or direct contact (including predation) with domestic dogs and cats (*Felis silvestris catus*), and subsequent transmission of their pathogens, potentially impacting puma populations. The transmission of infectious diseases from domestic dogs and cats have been demonstrated for other Chilean wild cats, facilitated by increased contact probability of human invasion into natural habitats and habitat fragmentation [69,70,71,72,73]. Cross-species transmission of pathogens as threats for puma populations have been studied in North America [74,75,76] and also for other wild felids [77,78,79]. However, to our knowledge, there are no disease studies at the wildland–urban interface in pumas in Chile, thus this potential threat is yet to be uncovered.

Coexistence and land-sharing must involve strategic regional planning and evidence-based management decisions, considering puma biology, landscape features, and human dimensions. A planning scheme should aim to identify areas with the highest human-puma overlap probabilities and try to reduce interactions between pumas and people, focusing on management, education, and landscape planning [49,54]. Strategies should also secure protected wild areas from where carnivore populations can expand and migrate, and protect corridors for dispersal among subpopulations to ensure long-term metapopulation persistence, especially those subpopulations affected by fragmentation or offtake by humans [56]. In our study area in the O’Higgins region, there is one small, protected area located in the intermediate depression (Las Palmas de Cocalán National Park; 3700 ha) and one large protected area at high elevations in the Andes (Rio de Los Cipreses National Reserve; 36,800 ha).

Improved public opinion and protective legislation have been proposed as crucial factors to enable the coexistence of large carnivores and people sharing the same landscapes outside protected areas [80]. Effective law enforcement is essential for coexistence, allowing a shift in social values, cultural acceptance, and favorable habitat changes [81,82]. Understanding the emotions involved in human-wildlife relationships [83,84] and the drivers of people’s tolerance towards those species perceived as dangerous [85,86] is needed. Records of *P. concolor* attacks on humans are scarce worldwide, and from a probabilistic perspective, they represent a minimal risk [87].

The current challenge is to develop and promote human–carnivore coexistence formulas that consider the local needs, resources, and cultural realities, allowing the sustainable balance between habitability and human development and the conservation of these carnivores [88], without cities becoming sinks for these species [89].

Our evidence of puma transit through or near human settlements without being commonly detected in the O’Higgins region suggests an implicit coexistence between human populations and wild carnivores, which still remains to be understood and assimilated by the local community. A growing interest of citizens in wildlife may be a good starting point, however, in the local communities of central Chile, much work still has to be advanced towards effective human–carnivore coexistence and the long-term persistence of all species.

## 5. Conclusions

Human–carnivore coexistence at the wildland–urban interface creates a number of management and conservation challenges. An interdisciplinary approach with social sciences professionals, wildlife managers, and other key actors is needed to develop successful coexistence formulas to allow the sustainable balance between human development and the long-term conservation of these carnivores sharing the same landscapes with people.

## Figures and Tables

**Table 1:** Records of *P. concolor* presence in O’Higgins Region, central Chile, obtained between May 2012 and July 2020. For each record, location, date, georeference (UTM WGS 84 H19), elevation (m.a.s.l.), and source and type of record are shown.

N°	Location	Date (m/d/y)	H19 E	H19 N	Elevation (m.a.s.l.)	Source of Record	Type of Record
1	Central Chacayes, Machalí	05-08-2012	373132	6198750	1116	SAG forensic analysis	Drowned puma corpse finding
2	Los Lirios, Rancagua	01-21-2013	335803	6210431	482	Citizen report	Video camera
3	Cortaderal, Machalí	02-14-2013	382327	6186473	1701	SAG field work	Live capture
4	Casa Piedra, Codegua	10-27-2014	364506	6232646	1897	SAG forensic analysis	Livestock attack
5	La Confluencia, San Fernando	12-12-2014	359295	6145326	1171	SAG forensic analysis	Livestock attack
6	Cerro Agujereado, Machalí	06-09-2015	365510	6208951	1040	Citizen report	Camera trap
7	Alto Huemul, San Fernando	09-25-2015	344185	6144160	1352	SAG field work	Sighting
8	Codegua, Chimbarongo	10-26-2015	322469	6147747	498	SAG forensic analysis	Livestock attack
9	El Baluarte, Rengo	11-24-2015	336950	6185055	535	SAG field work	Sighting
10	Glaciar Universidad, San Fernando	11-29-2015	375233	6156787	2414	Citizen report	Sighting
11	Central Chacayes, Machalí	12-07-2015	368219	6204112	1104	SAG forensic analysis	Drowned puma corpse finding
12	Puma Lodge, Machalí	12-30-2015	378698	6196868	1330	Citizen report	Sighting
13	Chapa Verde, Machalí	01-19-2016	367799	6231552	2324	SAG field work	Corpse finding
14	Cajón Río Blanco, Machalí	02-17-2016	377758	6213110	1685	SAG field work	Sighting
15	Cerrito San Juan, Machalí	07-26-2016	348847	6216399	623	SAG field work	Sighting
16	Los Peumos, RN Cipreses, Machalí	09-13-2016	366325	6202997	1210	Citizen report	Camera trap
17	Sierra Nevada, Machalí	12-22-2016	365496	6210477	903	SAG forensic analysis	Livestock attack
18	Alto Huemul, San Fernando	01-05-2017	349447	6133998	1807	SAG field work	Corpse finding
19	Chacayes, Machalí	01-22-2017	365077	6208430	885	SAG forensic analysis	Livestock attack
20	Central Chacayes, Machalí	02-21-2017	372176	6199283	1090	Citizen report	Sighting
21	La Correana, San Fernando	04-09-2017	360809	6136760	1409	SAG forensic analysis	Livestock attack
22	La Leonera, Codegua	06-01-2017	353359	6231408	724	Citizen report	Photograph
23	Los Petriles, Chimbarongo	09-02-2017	324072	6153298	436	SAG forensic analysis	Livestock attack
24	Cajón Portillo, San Fernando	10-28-2017	367890	6152761	1532	SAG field work	Sighting
25	La Rufina, San Fernando	10-29-2017	341114	6155275	794	SAG forensic analysis	Livestock attack
26	Haras Sauzal, Machalí	11-27-2017	352144	6207659	711	Police report	Sighting
27	Las Cayanas, Machalí	12-24-2017	379438	6211289	1639	SAG forensic analysis	Livestock attack
28	Cruce Alhué, Las Cabras	12-26-2017	282227	6225842	132	Citizen report	Corpse finding
29	Hotel La Leonera, Codegua	03-24-2018	354969	6232797	753	SAG forensic analysis	Livestock attack
30	La Polcura, Navidad	03-28-2018	228788	6228110	210	Citizen report	Photograph
31	Fundo Las Nieves, Rengo	04-04-2018	347370	6180227	851	SAG field work	Sighting
32	Embalse Cauquenes, Requinoa	08-06-2018	345620	6203567	797	Citizen report	Camera trap
33	Panilonco, Pichilemu	08-07-2018	230263	6201355	260	Citizen report	Photograph
34	Los Maquis, Pelequen	10-23-2018	331012	6184593	387	Citizen report	Sighting
35	Agua Buena, San Fernando	11-29-2018	333353	6165216	608	SAG forensic analysis	Livestock attack
36	La Pimpinela, Requinoa	12-11-2018	342374	6197274	490	Citizen report	Sighting
37	San Juan de Sierra, Chimbarongo	12-28-2018	324903	6157789	448	Citizen report	Sighting
38	Quebrada Santa Clara, Machalí	01-24-2019	371512	6242481	2725	SAG field work	Camera trap
39	Picarquín, Mostazal	02-13-2020	349266	6241144	586	SAG forensic analysis	Livestock attack
40	Tranque Barahona, Machalí	02-27-2019	360132	6227026	1582	Citizen report	Sighting
41	La Matancilla, San Fernando	03-04-2019	341469	6153015	962	SAG forensic analysis	Livestock attack
42	Camino Central Chacayes, Machalí	07-19-2019	365874	6207190	992	Citizen report	Photograph
43	Río Damas, San Fernando	10-02-2019	377486	6137038	2401	SAG field work	Camera trap
44	Maitenes, Machalí	12-17-2019	358206	6220093	1152	Citizen report	Video camera
45	Embalse Colihues, Requinoa	02-12-2020	344301	6208833	655	Citizen report	Camera trap
46	Termas del Flaco, San Fernando	03-02-2020	367279	6131495	1735	SAG forensic analysis	Livestock attack
47	Estero Los Leones, Requinoa	04-10-2020	347988	6203626	724	Citizen report	Camera trap
48	San Fernando	04-26-2020	318357	6169040	356	Citizen report	Video camera
49	Lago Rapel, Las Cabras	06-26-2020	272202	6216714	167	Citizen report	Sighting
50	Tranque Barahona, Machalí	07-06-2020	359558	6226429	1598	Citizen report	Camera trap
51	Panilonco, Pichilemu	07-29-2020	227101	6207978	180	Citizen report	Video camera

## Data Availability

The data presented in this study are available in Table 1.
